# Hypertension and gut microbial hydrogenases: a comparison of hydrogen metabolism and etiology

**DOI:** 10.4103/mgr.MEDGASRES-D-25-00128

**Published:** 2026-07-23

**Authors:** Yang Yi, Fei Xie, Chao Xia, Junyu Li, Pengxiang Zhao, Mengyu Liu, Xuemei Ma, Jinfeng Chen

**Affiliations:** 1 College of Chemistry and Life Science, Beijing University of Technology, Beijing, China; 2 Key Laboratory of Carcinogenesis and Translational Research (Ministry of Education/Beijing), Department of Thoracic Surgery II, Peking University Cancer Hospital & Institute, Beijing, China

**Keywords:** acetate metabolism, biomarkers, electron bifurcation, gut microbiota, hydrogen metabolism, hydrogenase, hypertension, Mendelian randomization, metabolic remodeling, metagenomics

## Abstract

Hypertension is a prevalent chronic condition and serves as a significant risk factor for numerous cardiovascular and cerebrovascular disorders. Gut microbiota dysbiosis has been considered to contribute to the pathogenesis of hypertension. It has been reported that a large majority of gut microbiota possess genes encoding hydrogenases. These hydrogenases are involved in the alteration of gut microbiota in non-infectious colitis, suggesting a potential link between microbial hydrogen metabolism and disease onset. This study aims to explore the relationship between hydrogenase expression patterns in the gut microbiome and the incidence of hypertension. In this study, publicly available gut microbiome metagenomic data were used to comprehensively analyze the expression patterns of hydrogenases in the gut microbiota of hypertensive patients. Compared with the control group, a 2.3-fold increase in electron bifurcating [FeFe] group A3 hydrogenases (*P* = 0.0299), a 55.6% decrease in [NiFe] group 1d hydrogenases (*P* = 0.0097), increased hydrogen-sensing hydrogenases and decreased hydrogen-uptake hydrogenases in the hypertension group. The main difference between the two groups is reflected in the abundance of [NiFe] hydrogenase subtypes. After eliminating the effects of factors such as age, sex, and lifestyle, significant differences in the abundance of [FeFe] group A3, [NiFe] group 1d, and [NiFe] group 1c were observed between the two groups, suggesting that these three indicators could serve as potential biomarkers for diagnosing the onset of hypertension. Additionally, Mendelian randomization analysis showed a protective effect of hydrogen metabolism against hypertension (odds ratio = 0.72, 95% confidence interval: 0.61–0.85, *P* < 0.001). Our study advances the understanding of microbiome-mediated mechanisms in hypertension by demonstrating an association between hydrogenase expression dynamics and blood pressure regulation, providing a foundation for future microbiome-based diagnostic and therapeutic strategies.

## Graphical abstract

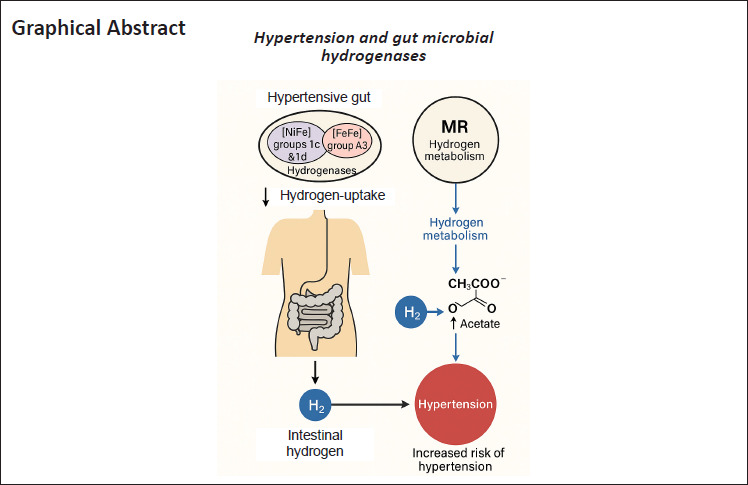

## Introduction

Hypertension, commonly known as high blood pressure, is one of the most prevalent non-communicable diseases worldwide. It affects more than 1.2 billion adults globally, with the burden increasing particularly in low- and middle-income countries.^1^ It is characterized by consistently elevated blood pressure levels, making it a leading risk factor for numerous cardiovascular and cerebrovascular disorders, including coronary heart disease, heart failure and stroke.^2^,^3^ The prevalence of hypertension and its related complications imposes a significant global public health burden, prompting extensive attention in medical research and healthcare initiatives.

Many studies have shown a connection between gut microbiome dysbiosis and high blood pressure, suggesting that imbalances in gut bacteria may contribute to hypertension development.^4^,^5^,^6^,^7^ Certain metabolites produced by gut bacteria, like short-chain fatty acids, are identified to affect blood pressure, indicating potential targets for therapeutic intervention.^8^,^9^ It has been shown that over 70% of gastrointestinal microbes in the Human Microbiome Project can metabolize H_2_, highlighting hydrogen cycling as a key energy-conservation mechanism in the gut with significant implications for human health and disease.^10^ There is already evidence that microbial hydrogen metabolism may affect human health. Campbell et al.^11^ found that high concentrations of colonic H_2_ stimulate the production of the anti-inflammatory metabolite butyrate, suggesting that H_2_ may serve as a regulator of fermentation in the human gut microbiome. Hughes et al.^12^ provide evidence that in response to gut inflammation, gut bacteria, especially Escherichia coli, increase hydrogenase production, facilitating hydrogen metabolism for a competitive survival advantage. This highlights the contribution of hydrogenases to alterations of the gut microbiota under disease conditions.

The ability of gut microbiota to metabolize hydrogen is attributed to their capability to synthesize hydrogenases. As a diverse group of metalloenzymes, hydrogenases catalyze the reversible conversion of hydrogen via splitting and formation.^11^ These enzymes can be categorized into three types ([FeFe] hydrogenase, [NiFe] hydrogenase, and [Fe] hydrogenase) based on the metal content in their active sites.^11^ Hydrogenases can also be classified into five categories, including uptake, evolving, bidirectional, bifurcating, and sensory enzymes, based on their predicted activities.^12^ In addition, Sondergaard et al.^13^ have classified hydrogenases into 3 major categories, 8 groups, and 38 subgroups based on their phylogenetic relationships. Among them, the [NiFe] hydrogenase category can be further divided into 4 groups and 29 sub-subgroups, the [FeFe] hydrogenase category into three groups and eight sub-subgroups, while the [Fe] hydrogenase category into only one group. Emerging studies have highlighted the crucial role of gut microbial hydrogen metabolism in host cardiovascular health, demonstrating its influence on systemic blood pressure regulation through modulation of the gut redox environment and short-chain fatty acid production.^14^,^15^,^16^,^17^ Alterations in gut microbiota composition, including changes in hydrogenase-expressing bacteria, have been linked to hypertension across different populations.^8^,^18^,^19^,^20^ Additionally, Mendelian randomization analyses have begun to clarify the potential causal relationships between gut microbial functions and hypertension, underscoring the importance of integrating host genetics with microbiome research.^21^,^22^ These insights emphasize the necessity to further explore hydrogenase expression dynamics within the gut microbiome in hypertension, particularly considering dietary and regional factors that may influence microbial composition and function.^23^,^24^ In summary, the diversity of hydrogenases in the gut microbiota endows them with different hydrogen metabolism capabilities. This diversity couples the hydrogen metabolism of different microbiota with energy metabolism, forming a relatively stable gut ecosystem.

Based on this framework, we hypothesize that: (1) Gut microbial hydrogenase expression profiles differ significantly between hypertensive and normotensive individuals; (2) These differential hydrogenase patterns directly modulate host blood pressure regulation; and (3) Host genetic factors influencing microbial hydrogen metabolism exhibit causal relationships with hypertension development.

To investigate these hypotheses, we pursued three sequential objectives: First, we performed systematic characterization of hydrogenase expression patterns in hypertensive versus control gut microbiomes using metagenomic profiling. Second, we identified specific hydrogenase subtypes associated with hypertension through comparative bioinformatics analysis. Third, we employed Mendelian randomization to establish causal links between microbial hydrogen metabolism genes and hypertension risk.

We hypothesize that gut microbial hydrogenase expression profiles differ significantly between hypertensive and normotensive individuals and that these differential hydrogenase patterns directly modulate host blood pressure regulation. We further hypothesize that host genetic factors influencing microbial hydrogen metabolism exhibit causal relationships with hypertension development. This study provides the first comprehensive analysis of hydrogenase expression dynamics in the hypertensive gut microbiome and aims to establish a genetic link between microbial hydrogen metabolism and blood pressure regulation.

## Methods

### Hydrogenase expression pattern analysis

**Figure 1:**
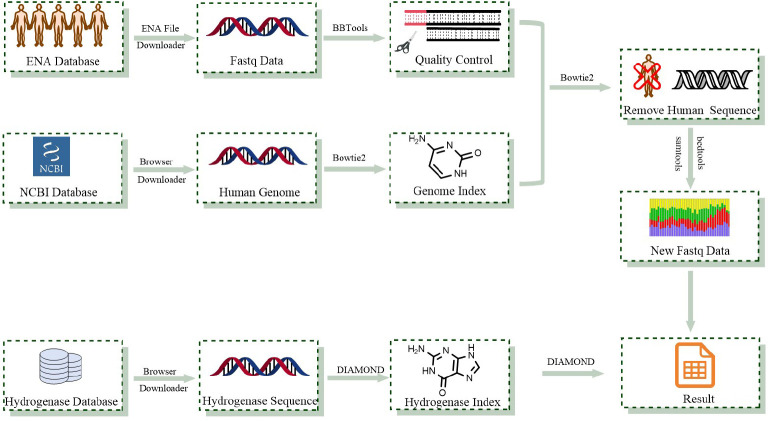
Metagenomic data processing and hydrogenase gene mapping process. This schematic illustrates the workflow for processing fecal shotgun metagenomic data and mapping hydrogenase genes. Sequencing data from human fecal samples, publicly available datasets (NCBI), and other metagenomic resources (ENA) were preprocessed and quality-controlled before alignment and annotation to identify hydrogenase genes. These annotated genes were then quantified and analyzed to assess differential abundance and subtype distribution, providing data for downstream statistical analysis. Created with ChemDraw Professional (version 22.0). ENA: European Nucleotide Archive; NCBI: National Center for Biotechnology Information.

To gain a comprehensive understanding of the expression patterns of hydrogenases in the gut microbiota of patients with hypertension, we utilized fecal shotgun metagenomic sequencing data derived from a published hypertension research project.^15^ The procedure for processing metagenomic raw data and mapping hydrogenase genes is illustrated in **Figure 1**. The raw data, obtained from the European Nucleotide Archive (https://www.ebi.ac.uk/ena/browser/home; project No. PRJEB21612, ENA accession No. ERP023883), represents a cohort study involving 60 hypertensive patients and 60 healthy controls. The shotgun metagenomic sequencing was performed on the Illumina HiSeq 3000 platform (Illumina, San Diego, CA, USA). Raw reads were processed using the BBTools package (BBDuk) of the BBMap software suite (version 38.90, Joint Genome Institute, Walnut Creek, CA, USA; https://jgi.doe.gov/data-and-tools/software-tools/bbtools/) to remove adapters and low-quality reads. We performed a rigorous three-step quality control process using BBDuk from the BBTools suite to ensure data reliability. In the first adapter-trimming step, we removed Illumina sequencing adapters and other known contaminants using a k-mer-based approach (*k* = 23, allowing 1 mismatch), which typically eliminates > 95% of adapter sequences while preserving > 90% of original reads. The second quality-trimming step employed a Phred quality threshold of 10 (Q10), removing bases with > 10% error probability from read ends, resulting in approximately 5–15% base removal per read. Final quality filtering discarded entire reads with mean quality scores below Q10, typically removing 5–20% of remaining reads depending on initial quality. Between each step, we assessed quality metrics using FastQC (v0.11.9), monitoring improvements in per-base quality scores (typically increasing by 5–15 Phred points), GC content stability (±2% variation), and sequence duplication levels (typically reduced by 10–30%). This stringent pipeline ensured only high-quality reads (Q10 average across all bases) proceeded to downstream analysis. Reads were then decontaminated against host genome using Bowtie2 (http://bowtie-bio.sourceforge.net/bowtie2/index.shtml).^25^ To evaluate the abundance of various hydrogenase categories in this metagenomic dataset, we performed Diamond BLAST on the clean and filtered reads against the HydDB hydrogenase database. This database, consisting of 3260 known sequences, was sourced from the College of Biosciences at Nash University and the Bioinformatics Research Center at Aarhus University.^11^

### Statistical analysis

Statistical analyses were performed using GraphPad Prism 9.0 (GraphPad Software, San Diego, CA, USA; www.graphpad.com) and R software (version 4.2.0; R Foundation for Statistical Computing, Vienna, Austria; https://www.r-project.org/). For comparisons between two groups, the non-parametric Mann–Whitney *U* test was used. For comparisons involving more than two groups, the Kruskal-Wallis test followed by Dunn’s *post hoc* test was applied. Statistical significance was set at *P* < 0.05. Data are presented as median and interquartile range unless otherwise specified. The differential abundance of each hydrogenase category was subsequently determined using DESeq2 (version 1.34.0; https://bioconductor.org/packages/release/bioc/html/DESeq2.html), with normalization to the total number of reads aligned to all the queried hydrogenasesn.^26^,^27^

### Mendelian randomization analysis

The Mendelian randomization process in the study leverages a hypertension dataset (accession No. GCST90038604) from the IEU Open GWAS database (https://gwas.mrcieu.ac.uk/),^28^ featuring 12,909 cases and 354,689 controls, drawn from a British population. The human reference genome sequence was downloaded from the National Center for Biotechnology Information (NCBI). Statistical analyses were implemented using the R platform with the “TwoSampleMR” package (version 0.5.6; https://mrcieu.github.io/TwoSampleMR/).^28^

**Figure 2:**
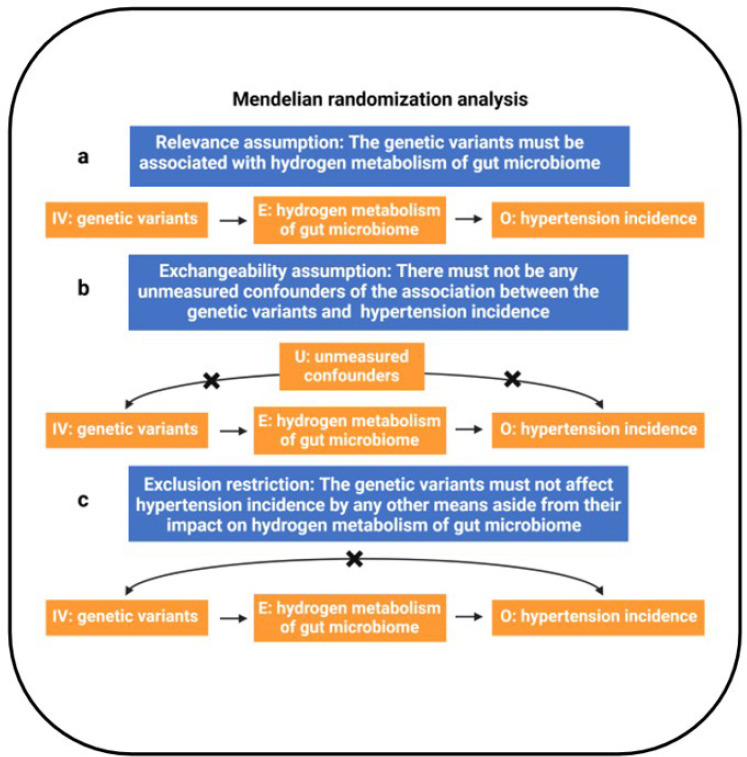
Integration of Mendelian randomization analytical framework Schematic representation of the key assumptions behind Mendelian randomization: relevance (a), exchangeability (b), and exclusion restriction (c), necessary for inferring causality. Created with ChemDraw Professional (version 22.0). E: Exposure; IV: instrumental variable; O: outcome; U: unmeasured confounder.

To elucidate the relationship between hydrogen metabolism by the gut microbiome and the incidence of hypertension, we adopted a Mendelian randomization approach. We selected genetic variants strongly associated with the exposure of interest—hydrogen metabolism in the gut microbiome—to serve as instrumental variables. We confirmed that the instrumental variables were significantly correlated with hydrogen metabolism, ensuring their relevance as predictors. We examined potential confounders to ensure the instrumental variables were independent of any such variables that might also affect hypertension incidence. We verified that the instrumental variables influenced the risk of hypertension only through their impact on hydrogen metabolism, without any other indirect pathways. Using these instrumental variables, we conducted regression analyses to determine the magnitude and significance of the association between hydrogen metabolism and hypertension (**Figure 2**).

## Results

### Hydrogenase distribution across taxonomic groups of the gut microbiota

**Figure 3:**
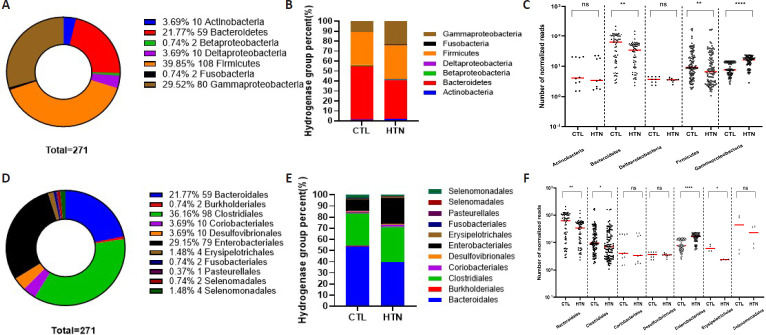
Expression patterns of hydrogenase sequences at the phylum and order levels. (A) The expression pattern of hydrogenases at the phylum level. (B) Proportional representation of hydrogenases at the phylum level in the control (CTL) group and hypertension (HTN) group. (C) Specific abundance changes of hydrogenases at the phylum level in CTL and HTN groups. (D) The expression pattern of hydrogenases at the order level. (E) Proportional representation of hydrogenases at the order level in CTL and HTN groups. (F) Specific abundance changes of hydrogenases at the order level in CTL and HTN groups, with orders having *n* < 4 removed. Each point represents the average number of reads mapped to specific hydrogenase sequences after normalization by a standard factor. Statistical analysis was conducted using the non-parametric Mann–Whitney *U* test, with error lines representing the interquartile range, *P < 0.05, **P < 0.01, ***P < 0.001.

The gut microbiome metagenomic data were aligned to the HydDB database, resulting in the identification of 271 mapped hydrogenase gene sequences. The identified hydrogenase sequences were distributed across seven bacterial phyla, specifically: Firmicutes (39.85%), Gammaproteobacteria (29.52%), and Bacteroidetes (21.77%), which collectively accounted for 91.14% of the total. The remaining phyla included Actinobacteria (3.69%), Deltaproteobacteria (3.69%), Betaproteobacteria (0.74%), and Fusobacteria (0.74%). Differences in hydrogenase abundance between hypertensive patients and controls were primarily observed in the phyla Firmicutes, Gammaproteobacteria, and Bacteroidetes. The abundance of hydrogenase significantly increased in the Firmicutes (*P* = 0.0062) and Bacteroidetes (*P* = 0.0067) phyla of the gut microbiome in hypertensive patients, while it markedly decreased (*P* < 0.0001) in the Gammaproteobacteria phylum. At the order level, hydrogenases were expressed in 11 orders, with primary expression observed in Clostridiales, Enterobacteriales, and Bacteroidales, accounting for 87.08% of the total. Differences in hydrogenase abundance between the hypertensive and normal groups were also primarily observed in these three orders. In hypertensive patients, the abundance of hydrogenase significantly increased in Bacteroidetes (*P* = 0.0062), Clostridiales (*P* = 0.0101), and Erysipelotrichale (*P* = 0.0286), while it significantly decreased (*P* < 0.0001) in the Enterobacteriales (**Figure 3**). These findings suggest that the expression pattern of hydrogenases in hypertensive patients has changed significantly, which may play a crucial role in microbial dysbiosis and ultimately influence the development of hypertension.

### Expression patterns of hydrogenases with different predicted activities

**Figure 4:**
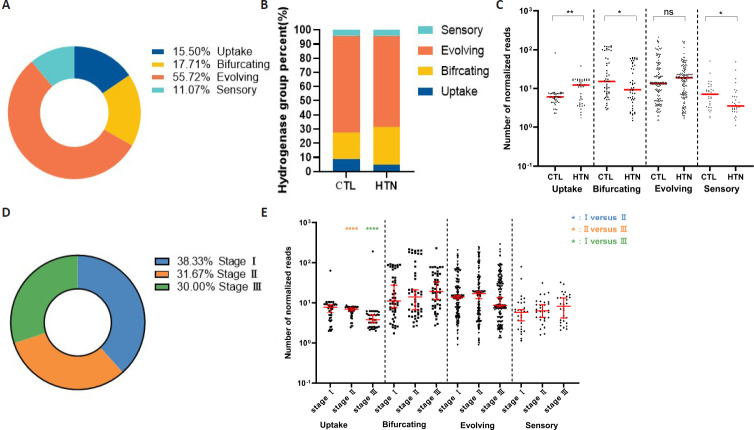
Expression patterns among hydrogenases exhibiting distinct predicted activities. (A) Proportions of hydrogenases with different predicted functions. (B) The proportions of four types of hydrogenases with distinct predicted activities in hypertensive patients and controls. (C) Comparison of the abundance of hydrogenases with four different predicted activities between hypertensive patients and controls. (D) The proportion of hypertensive patients in different stages. (E) Comparative analysis of the abundance of four hydrogenases with distinct predicted activities in hypertensive patients at different stages. Each point represents the average number of normalized reads mapped to specific hydrogenase sequences, with statistical analysis using the Mann–Whitney non-parametric test. Error lines correspond to the interquartile range, *P < 0.05, **P < 0.01, ***P < 0.001. CTL: Control; HTN: hypertension.

Reads were aligned with predicted hydrogenase activities in the HydDB hydrogenase database, resulting in the identification of four types of hydrogenases with distinct predicted functions: evolving (55.72%), uptake (15.50%), bifurcating (17.71%), and sensory (11.07%) hydrogenases (**Figure 4A**). There was a significantly higher abundance of reads that aligned to predicted bifurcating (*P* = 0.299) and sensory (*P* = 0.0430) hydrogenases in the hypertension group, while the abundance of predicted uptake hydrogenase genes significantly decreased (*P* = 0.033). No significant difference was observed in the abundance of the reads that aligned to the evolving hydrogenases (**Figure 4B** and **C**). Further analysis was conducted to investigate the relationship between the abundance of hydrogenases with distinct predicted activities and hypertension stages. According to guidelines from the Association of Anaesthetists of Great Britain and Ireland and the British Hypertension Society,^29^ 60 hypertensive patients were categorized into three stages: Stage I (140/90 to 159/99 mmHg), Stage II (160/100 to 179/109 mmHg), and Stage III (exceeding 180/110 mmHg). Among the four types of hydrogenases with predicted activity, only the abundance of uptake hydrogenase shows a significant correlation with hypertension staging. Specifically, as hypertension staging increases, the abundance of uptake hydrogenase gradually decreases. Stage II patients exhibit notably higher levels of this hydrogenase (*P* < 0.0001) compared with Stage III patients, while Stage I patients show a non-significant increase relative to Stage II patients (**Figure 4D** and **E**).

### Expression pattern of different groups of hydrogenases based on phylogenetic classification

**Figure 5:**
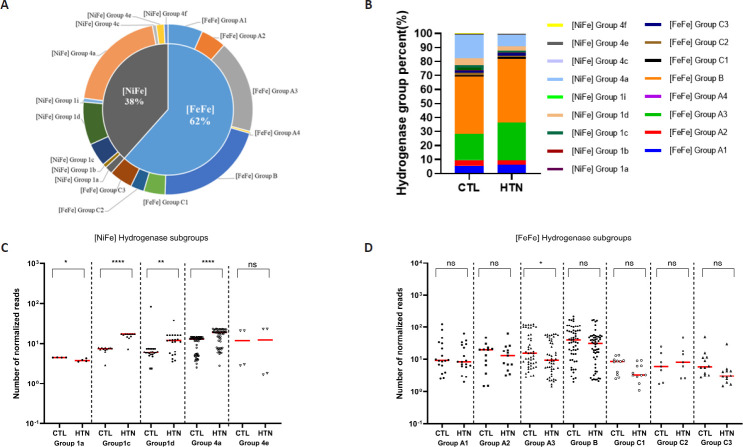
expression patterns among hydrogenases based on phylogenetic classification (A) Proportions of different hydrogenase subtypes based on phylogenetic classification. (B) Proportions of different hydrogenase subtypes in hypertensive patients and controls. (C) Comparison of the abundance of various subgroups of [NiFe] hydrogenases between hypertensive patients and controls. (D) Comparison of the abundance of various subgroups of [FeFe] hydrogenases between hypertensive patients and controls. Subtypes with *n* < 4 have been removed. Statistical analysis was performed using the non-parametric Mann–Whitney *U* test, with error bars representing the interquartile range, *P < 0.05, **P < 0.01, ***P < 0.001. CTL: Control; HTN: hypertension.

As shown in **Figure 5A**, the aligned reads comprise two types of hydrogenases: namely [FeFe] hydrogenases (62%) and [NiFe] hydrogenases (38%). The proportions of the various subgroups of [FeFe] and [NiFe] hydrogenases in the hypertension group and the control group are shown in **Figure 5B**. Among [NiFe] hydrogenases, the abundances of Group 1c (*P* < 0.0001), Group 1d (*P* = 0.0097), and Group 4a (*P* < 0.0001) were significantly decreased in the hypertensive patient group, while the abundance of Group 1a (*P* = 0.0286) was significantly increased (**Figure 5C**). There was no significant difference in the abundance of most [FeFe] hydrogenases between hypertensive patients and controls, except for [FeFe] group A3, which showed a significantly higher abundance (*P* = 0.0299) in hypertensive patients compared to the control group (**Figure 5B** and **C**).

### An increase in the abundance of [FeFe] group A3 and a decrease in [NiFe] group 1d and [NiFe] group 1c within the gut microbiota are markers of hypertension

**Figure 6:**
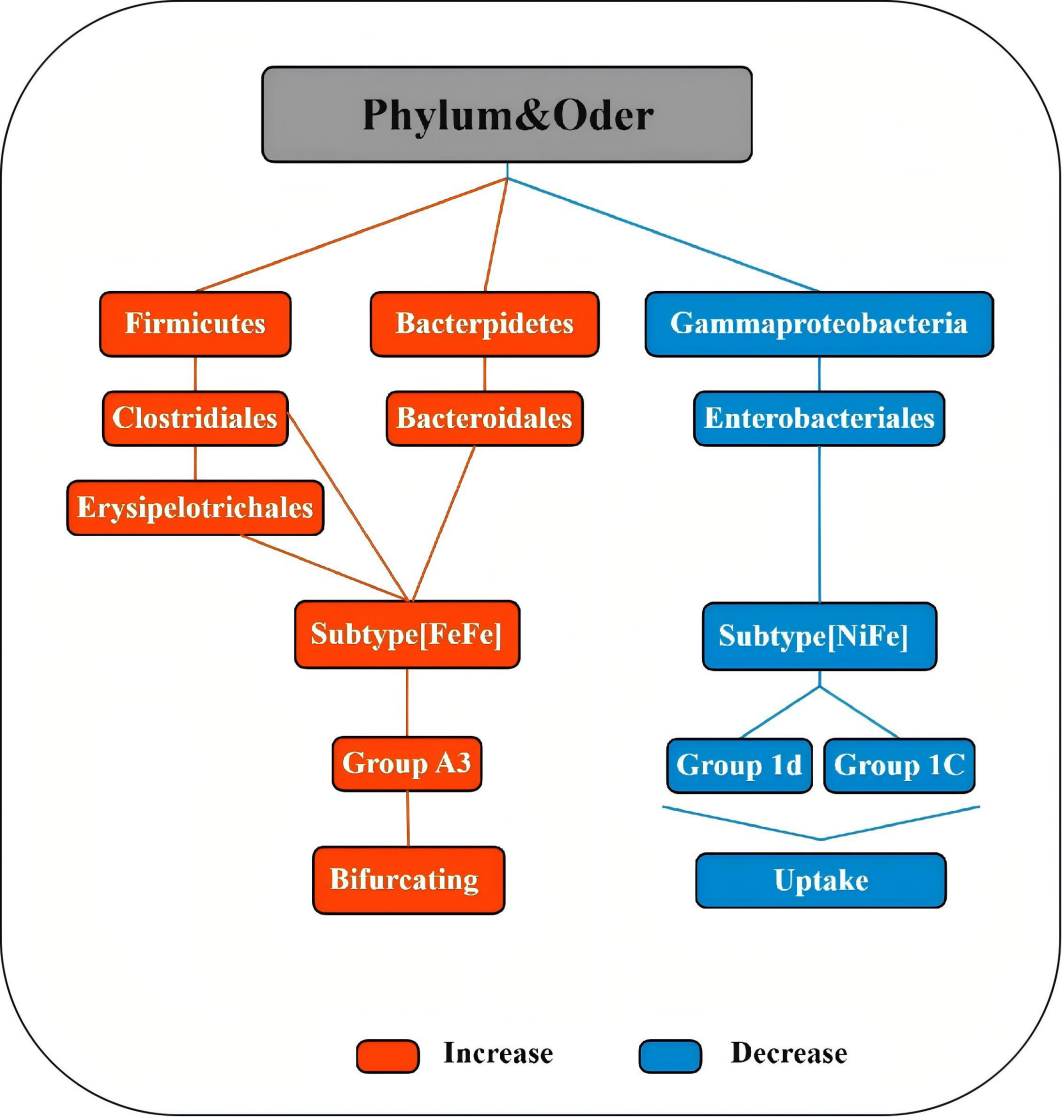
The relationship network of hydrogenase abundance in the gut microbiota between hypertensive patients and healthy individuals. Created with ChemDraw Professional (version 22.0).

As mentioned above, we examined the types, functions, and subtypes of hydrogenase-producing bacterial species in the gut microbiome of hypertensive patients. Considering the interconnections between various factors, we summarized the results and found that although the types of bacterial species that increased in the gut microbiome of hypertensive patients varied—predominantly from the phyla Bacteroidetes and Firmicutes—the abundance of [FeFe] hydrogenases was significantly elevated in each phylum. This elevation was mainly characterized by high expression of the group A3 subtype, all of which are electron-bifurcating hydrogenases. Conversely, the expression of [NiFe] hydrogenases was significantly reduced, corresponding to subtypes group 1d and group 1c (**Figure 6**). Although comparisons of hydrogenase functions revealed that hydrogen-sensing hydrogenases were also highly expressed in the gut microbiome of hypertensive patients, correlation analysis of the three factors showed that the subtypes associated with hydrogen-sensing were all [FeFe] hydrogenases, with no significant differences in groups C1, C2, and C3. This could be due to other non-critical factors such as age, sex, and lifestyle habits, and indeed, our analysis of these factors revealed differences in the expression of hydrogen-sensing hydrogenases (**Additional Figures 1–3**).

### Mendelian randomization analysis reveals a causal relationship between hydrogen metabolism in gut microbiota and the onset of hypertension

**Figure 7:**
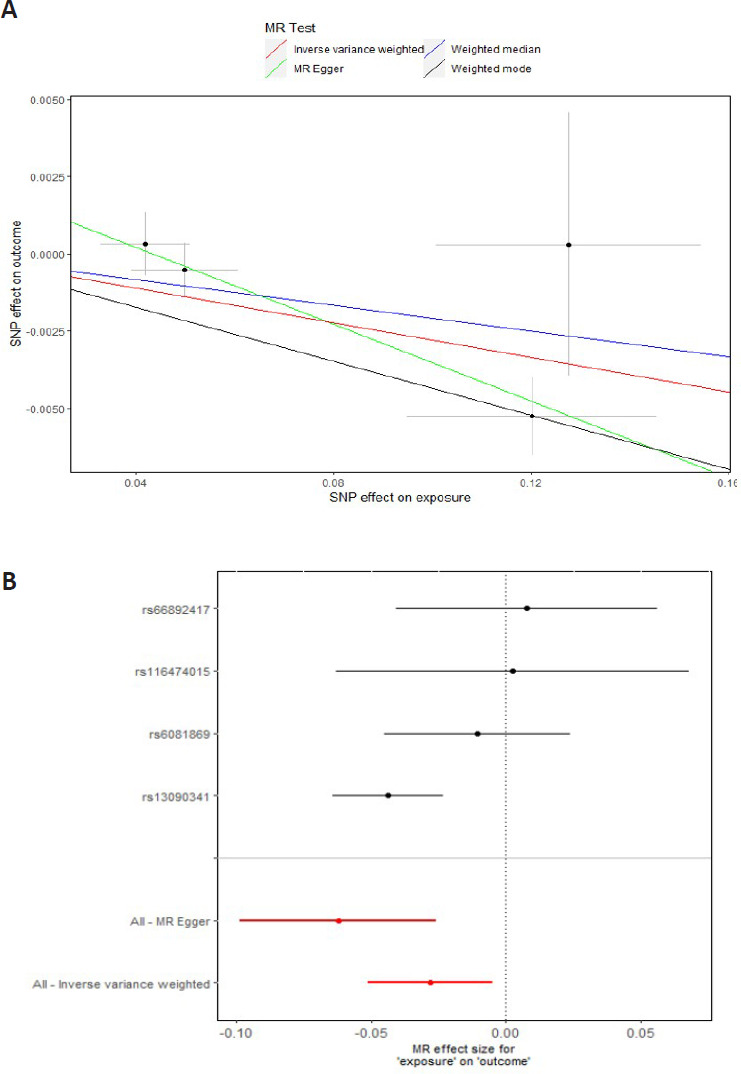
Integration of Mendelian randomization analytical results. (A) Scatter plot illustrating the relationship between genetic variants associated with hydrogen metabolism of gut microbiome (instrumental variables) and hypertension incidence. (B) Forest plot summarizing the effect sizes derived from different Mendelian randomization methods, indicating the influence of gut microbiome on hypertension risk. MR: Mendelian randomization; SNP: single nucleotide polymorphisms.

Initially, we identified 26 single nucleotide polymorphisms significantly associated with hydrogen metabolism in the gut microbiome in a discovery cohort of 1539 individuals,^30^ marked by a statistical significance threshold of *P* < 1 × 10^–6^. This step highlighted a genetic propensity that could impact the metabolic functions of the gut microbiota. Furthermore, we utilized the extensive hypertension dataset GCST90038604, which contains over 9.5 million single nucleotide polymorphisms associated with the disease incidence. After accounting for linkage disequilibrium, we focused our analysis on four single nucleotide polymorphisms closely related to the disease. Employing various Mendelian randomization methods, including inverse variance weighted, Mendelian randomization-Egger, weighted median, and weighted mode, we validated the direction and strength of the causal relationship. The results indicated a consistent negative correlation between hydrogen metabolism and hypertension across different Mendelian randomization methods (**Figure 7A**). The primary inverse-variance weighted analysis demonstrated that each standard deviation increase in genetically predicted hydrogen metabolism activity was associated with a 28% reduction in the risk of hypertension (odds ratio [OR] = 0.72, 95% confidence interval [CI] 0.61–0.85; *P* = 0.0002). Sensitivity analyses yielded directionally consistent results: weighted median estimator (OR = 0.75, 95% CI 0.62–0.91; *P* = 0.003), Mendelian randomization-Egger regression (OR = 0.69, 95% CI 0.51–0.93; *P* = 0.014), and weighted mode estimator (OR = 0.71, 95% CI 0.55–0.92; *P* = 0.009). The Mendelian randomization-Egger intercept test showed no evidence of directional pleiotropy (intercept = –0.008, *P* = 0.38), and leave-one-out analyses confirmed that no single nucleotide polymorphism was driving the observed associations (**Figure 7B**). The convergence of results from multiple Mendelian randomization methods enhances the likelihood of a causal relationship.

## Discussion

In the analysis of hydrogenase functions, we found that, compared to other countries’ metagenomic databases, bidirectional hydrogenases were not expressed in our dataset. This might be related to differences in sequencing methods, depth, or regional dietary habits. Four types of functional hydrogenases were expressed: hydrogen-uptake enzymes, hydrogen-evolving enzymes, electron-bifurcating hydrogenases, and hydrogen-sensing hydrogenases. Hydrogen-uptake enzymes, typically found in bacteria and archaea, use hydrogen as the primary electron donor and efficiently remove it from the environment, requiring adenosine triphosphate-derived energy for hydrogen oxidation.^31^,^32^ In hypertensive patients, the abundance of these enzymes was reduced and stage-dependent, indicating lower hydrogen utilization. However, in subsequent analyses, hydrogen-uptake hydrogenases did not remain a focus, while electron-bifurcating hydrogenases were more representative. The substrate differences between hydrogen-uptake enzymes (using fumarate, producing succinate) and electron-bifurcating hydrogenases (using carbon dioxide, producing acetate)^10^ may explain the disappearance of these variations in later analyses. Additionally, as hydrogen-uptake hydrogenases ([NiFe]) are less prevalent than [FeFe] hydrogenases in the human gut, they may be filtered out during analysis, and separate screening would be required, which was beyond this study’s focus.

[FeFe] group A3 hydrogenases, involved in electron bifurcation, are associated with acetate production via reactions with carbon dioxide and hydrogen in Clostridia.^10^ Our data suggested an increase in [FeFe] group A3 hydrogenases in hypertensive individuals, indicating higher acetate levels. Although acetate and similar short-chain fatty acids can lower blood pressure,^16^,^17^,^33^,^34^,^35^ a longitudinal study showed that hypertensive patients had higher stool short-chain fatty acid levels, correlating with higher blood pressure.^36^ This suggests that simply increasing gut acetate may not effectively control blood pressure and could even be counterproductive.

Several factors may explain this contradiction. Regional and dietary differences could influence the effects of acetate, as our Chinese cohort may differ from Western populations in dietary fiber intake and microbial composition. Additionally, while [FeFe] A3 hydrogenases promote acetate production, reduced [NiFe] hydrogenases may impair hydrogen utilization, leading to hydrogen accumulation and pro-inflammatory metabolite production. The vasoprotective effects of acetate could also be offset by chronic inflammation during hypertension progression. Unlike other studies, we accounted for potential confounders, including sex, age, and smoking, strengthening the reliability of our gene selection.

Our Mendelian randomization analysis indicated that enhanced hydrogen metabolism may reduce hypertension risk,^37^ aligning with our hydrogenase findings and suggesting that hydrogenase-containing gut microbiota may influence hypertension development. These findings reflect remodeling of the gut microbial metabolic network, with the potential for microbial hydrogenase subtypes to serve as biomarkers and therapeutic targets in hypertension. However, further validation across diverse populations is needed to establish standardized cutoff values and evaluate clinical applicability.

Despite the strengths of this study, several limitations should be acknowledged. First, the gut metagenomic data were obtained from a single publicly available cohort, which may not fully capture population heterogeneity, especially across different geographic, ethnic, and dietary backgrounds. Second, although we performed rigorous bioinformatic filtering and normalization, the use of reference-based hydrogenase databases may limit the identification of novel or unclassified hydrogenase genes. Third, shotgun metagenomic sequencing, while powerful, does not directly reflect gene expression or enzyme activity, and functional inferences rely on gene presence and abundance rather than confirmed metabolic outputs. Additionally, no independent experimental validation or animal models were employed to corroborate the role of hydrogenase subtypes in blood pressure regulation. Finally, the Mendelian randomization analysis was restricted to genetic associations in a European population, which may limit its generalizability to other ethnic groups, including our primary dataset from a Chinese cohort. Future research with multi-ethnic, longitudinal, and experimental validation will be necessary to confirm and expand upon these findings.

In conclusion, this study advances the understanding of microbiome-mediated mechanisms in hypertension by demonstrating an association between hydrogenase expression dynamics and blood pressure regulation, providing a foundation for future microbiome-based diagnostic and therapeutic strategies.

## Additional files:

***Additional Figure 1:*** *Proportions of different age groups and specific abundance changes of various types of hydrogenases.*

Additional Figure 1 Proportions of different age groups and specific abundance changes of various types of hydrogenases.
(A) Overall age distribution of the samples. (B) Age distribution in the normal group. (C) Age distribution in the hypertensive group. (D–G) Changes in the abundance of hydrogen-consuming, electronbifurcating, hydrogen-releasing, and inducible hydrogenases across different age groups. Statistical analyses for the CTL and HTN groups were performed using the Mann-Whitney test, while within-group analyses utilized the Kruskal-Wallis test, with error bars representing the interquartile range, ^*^P < 0.05, ^**^P < 0.01, ^***^P < 0.001. CTL: Control; HTN: hypertension.

***Additional Figure 2:*** *Sex distribution and specific abundance changes of various types of hydrogenases.*

Additional Figure 2 Sex distribution and specific abundance changes of various types of hydrogenases.
(A) Overall sex distribution of the samples. (B) Sex distribution in the normal group. (C) Sex distribution in hypertensive patients. (D) Specific abundance changes of hydrogen-uptake hydrogenases in different sexes. (E) Specific abundance changes of electron-bifurcating hydrogenases in different sexes. (F) Specific abundance changes of hydrogen-evolving hydrogenases in different sexes. (G) Specific abundance changes of sensory hydrogenases in different sexes. For the statistical analysis in the CTL) and HTN groups, the non-parametric Mann-Whitney *U* test was used. Within-group statistical analyses in the CTL or HTN groups were conducted using the Kruskal–Wallis test, with error bars representing the interquartile range, ^*^P < 0.05, ^**^P < 0.01, ^***^P < 0.001. CTL: Control; HTN: hypertension.

***Additional Figure 3:*** *Sample smoking status and specific abundance changes of different types of hydrogenases.*

Additional Figure 3 Sample smoking status and specific abundance changes of different types of hydrogenases.
(A) Overall smoking proportion in the sample. (B) Smoking distribution in the normal group. (C) Smoking distribution among hypertensive patients. (D) Impact of smoking on the specific abundance of hydrogen-uptake hydrogenases. (E) Impact of smoking on the specific abundance of electron-bifurcating hydrogenases. (F) Impact of smoking on the specific abundance of hydrogen-evolving hydrogenases. (G) Impact of smoking on the specific abundance of sensory hydrogenases. For statistical analysis in the CTL and HTN groups, the non-parametric Mann-Whitney *U* test was used. Within-group statistical analyses in the CTL or HTN groups were conducted using the Kruskal-Wallis test, with error bars representing the interquartile range, ^*^P < 0.05, ^**^P < 0.01, ^***^P < 0.001. CTL: Control; HTN: hypertension.

## Data Availability

*All data generated or analyzed in this study are included in this published article and its additional files.*
